# Gamma Radiation-Induced Disruption of Cellular Junctions in HUVECs Is Mediated through Affecting MAPK/NF-*κ*B Inflammatory Pathways

**DOI:** 10.1155/2019/1486232

**Published:** 2019-08-04

**Authors:** H. Wang, R. Chandra Segaran, L. Y. Chan, Aref A. M. Aladresi, A. Chinnathambi, S. A. Alharbi, G. Sethi, F. R. Tang

**Affiliations:** ^1^Radiobiology Research Laboratory, Singapore Nuclear Research and Safety Initiative, National University of Singapore, Singapore 138602; ^2^Department of Botany and Microbiology, College of Science, King Saud University, Riyadh 11451, Saudi Arabia; ^3^Department of Pharmacology, Yong Loo Lin School of Medicine, National University of Singapore, Singapore 117600

## Abstract

Ionizing radiation-induced cardiovascular diseases (CVDs) have been well documented. However, the mechanisms of CVD genesis are still not fully understood. In this study, human umbilical vein endothelial cells (HUVECs) were exposed to gamma irradiation at different doses ranging from 0.2 Gy to 5 Gy. Cell viability, migration ability, permeability, oxidative and nitrosative stresses, inflammation, and nuclear factor kappa-light-chain-enhancer of activated B cell (NF-*κ*B) pathway activation were evaluated postirradiation. It was found that gamma irradiation at doses ranging from 0.5 Gy to 5 Gy inhibited the migration ability of HUVECs without any significant effects on cell viability at 6 h and 24 h postirradiation. The decreased transendothelial electrical resistance (TEER), increased permeability, and disruption of cellular junctions were observed in HUVECs after gamma irradiation accompanied by the lower levels of junction-related proteins such as ZO-1, occludin, vascular endothelial- (VE-) cadherin, and connexin 40. The enhanced oxidative and nitrosative stresses, e.g., ROS and NO_2_^−^ levels and inflammatory cytokines IL-6 and TNF-*α* were demonstrated in HUVECs after gamma irradiation. Western blot results showed that protein levels of mitogen-activated protein kinase (MAPK) pathway molecules p38, p53, p21, and p27 increased after gamma irradiation, which further induced the activation of the NF-*κ*B pathway. BAY 11-7085, an inhibitor of NF-*κ*B activation, was demonstrated to partially block the effects of gamma radiation in HUVECs examined by TEER and FITC-dextran permeability assay. We therefore concluded that the gamma irradiation-induced disruption of cellular junctions in HUVECs was through the inflammatory MAPK/NF-*κ*B signaling pathway.

## 1. Introduction

Cardiovascular disease (CVD) has been considered as one of the noncancer health risks associated with ionizing radiation. Recent studies suggested a causality between the development of cardiovascular disease and radiation exposure [1, 2]. Endothelial cells, as the most sensitive cell type in the vascular system, are the critical targets in cardiovascular damage induced by radiation, which plays a key role in the development of vascular pathologies, such as endothelial barrier damage and permeability changes [3, 4], premature senescence [5, 6], angiogenic defects [7], and the development of atherosclerosis [8–10]. The endothelial integrity is closely associated with the barrier functions of intercellular junctions. An increased permeability in the vascular system is a common consequence after radiation exposure and is believed to be an important element in the development of radiation-induced CVD complications [3].

Three major types of intercellular junctions exist in vertebrates: tight junctions, adherens junctions, and gap junctions [11]. Occludin and claudin-5 are the fundamental transmembrane proteins in the formation of tight junctions and connect to the cytoskeleton through the ZO family [12]. Adherens junctions are formed by cadherins linked to the cytoskeleton by catenin family proteins, and vascular endothelial- (VE-) cadherin is an endothelial specific adherens junction protein [13]. Connexins compose the gap junction channels [14], and connexin 40 was found highly expressed in endothelial cells [15, 16]. There are increasing evidences indicating the altered intercellular junction function induced by gamma radiation exposure [11, 12, 17].

Gamma rays, as a kind of ionizing radiation, induce the water radiolysis inside the body and produce the damage through free radicals, including *reactive oxygen species* (*ROS*) and nitric oxide (NO) [18, 19]. Oxidative and nitrosative stresses are potent pathogenic mechanisms in lipid peroxidation, inflammation, DNA damage, and cellular dysfunction. Inflammation is a key factor in the development of atherosclerosis [8]. Under pathological conditions, inflammatory mediators can compromise barrier function, which is correlated with active cytoskeletal remodelling and gap formation between adjacent endothelial cells [3, 20, 21]. Although it is well accepted that inflammatory and oxidative and nitrosative stress pathways exert a detrimental effect on endothelial barrier integrity and function [22], the underlying molecular mechanisms remain unclear.

Gamma irradiation activated nuclear factor kappa-light-chain-enhancer of activated B cell (NF-*κ*B) signaling pathways by multiple mechanisms [19, 23–25]. The regulation of the NF-*κ*B pathway is involved in the survival and death of cells exposed to gamma radiation. Our previous studies indicated that gamma radiation exposure time- and dose-dependently enhanced NF-*κ*B DNA-binding activities in HaCaT cells [18, 19]. The activation of the NF-*κ*B pathway was also observed in endothelial cells after exposure to gamma radiation [26], X-ray [9], or UVB [27]. Therefore, we hypothesized that gamma irradiation-induced inflammation and oxidative and nitrosative stresses might cause endothelial barrier damage through the regulation of the NF-*κ*B signaling cascade. To test our hypothesis, we systematically examined the effects of gamma irradiation with different doses on the mobility and permeability of HUVECs and explored the possible underlying molecular mechanisms via the mitogen-activated protein kinase (MAPK)/NF-*κ*B pathway. The effects of the NF-*κ*B activation inhibitor were also investigated.

## 2. Methods

### 2.1. Reagents

The following reagents were obtained: EGM^TM-2^ Endothelial Cell Growth Basal Medium-2 from Lonza, Basel, Switzerland; 3-(4,5-dimethylthiazol-2-yl)-2,5-diphenyltetrazolium bromide (MTT), dimethyl sulfoxide (DMSO), paraformaldehyde, Triton™ X-100, Tris, glycine, sodium dodecyl sulfate (SDS), CelLytic™ mammalian cell lysis/extraction reagent, bovine serum albumin (BSA), and 4 kDa FITC-dextran from Sigma-Aldrich (St. Louis, MO, USA); rat tail collagen type I from Merck (Singapore); 2′,7′-dichlorofluorescin diacetate (DCFDA) cellular ROS detection assay kit from Abcam (Cambridge, MA, USA); mouse anti-occludin antibody, Superclonal™ goat anti-mouse IgG (H+L) Alexa Fluor 488 and Alexa Fluor 555, and ProLong™ Gold Antifade Mountant with DAPI from Invitrogen (Carlsbad, CA, USA); Griess Reagent Kit from Promega (Madison, WI, USA); ELISA kits for interleukin-6 (IL-6) and tumor necrosis factor alpha (TNF-*α*) from R&D Systems (Minneapolis, MN, USA); antibodies against ZO-1, endothelial NOS (eNOS), p38 MAPK, phospho-p38 MAPK (Thr180/Tyr182), p27, phospho-p65 (Ser 536), p65, and *β*-actin from Cell Signaling Technology (Beverly, MA, USA); antibodies against claudin-5 and connexin 40 (Cx40/GJA5) from Abcam (Singapore); an inhibitor of NF-*κ*B activation BAY 11-7085, antibodies against VE-cadherin, p53, p21, horseradish peroxidase- (HRP-) conjugated goat anti-rabbit antibodies, and HRP-conjugated goat anti-mouse antibodies from Santa Cruz Biotechnology (Santa Cruz, CA, USA); BCA assay kit and nitrocellulose membrane from Bio-Rad (Hercules, CA, USA); and Amersham ECL Prime Western Blotting Detection Reagent from GE Healthcare (Buckinghamshire, UK). The following kits were also obtained: nuclear extract kit and TransAM™ NF-*κ*B Transcription Factor Assay kits from Active Motif (CA, USA); Halt™ Protease and Phosphatase Inhibitor Cocktail (100x), Maxima First Strand cDNA Synthesis Kit, and Maxima SYBR Green/ROX qPCR Master Mix from Thermo Fisher Scientific (Singapore); and RNeasy Mini Kit from QIAGEN (Singapore).

### 2.2. Cell Culture

HUVECs were purchased from Lonza (Basel, Switzerland) and cultured in EBM™-2 medium containing 2% fetal bovine serum (FBS), 1‰ ascorbic acid, basic fibroblast growth factor (bFGF), vascular endothelial growth factor (VEGF), epidermal growth factor (EGF), insulin-like growth factor-1 (IGF-1), hydrocortisone, heparin, and gentamicin sulfate amphotericin in a humidified atmosphere with 5% CO_2_ at 37°C. Cells were passaged every three to four days, and HUVECs at the 4th to 8th passages were employed this study.

### 2.3. Irradiation Procedure

For different experiments, HUVECs were seeded in 96-well plates, Culture-Insert 2 wells, 8W10E+ arrays, cell culture chamber slides, Transwell inserts, 75 cm^2^ cell culture flasks, or 60 mm^2^ cell culture dishes and grown to confluence before being irradiated with the Irradiator BIOBEAM 8000 (Gamma-Service Medical GmbH, Leipzig, Germany) with Cs-137 as radioactive sources. Different radiation doses were applied for the dose-response curve. Groups without any radiation exposure served as the controls. HUVECs were harvested at 6 h or 24 h after irradiation and subjected to different assays.

### 2.4. Cell Viability Assay

The viability of HUVECs after gamma irradiation was examined by colorimetric MTT assay. The method was based on the cleavage of MTT, a yellow tetrazolium salt, to purple formazan crystals by metabolically active cells. The formazan crystals were solubilized and then quantified spectrophotometrically. Briefly, HUVECs were seeded in 96-well plates, irradiated with different doses, ranging from 0.2 Gy to 5 Gy, and then incubated for 6 or 24 hours. A final concentration of 0.5 mg/ml MTT was added to each well and incubated at 37°C for 4 h. After the removal of the medium, HUVECs were washed with PBS. 100 *μ*l DMSO was added into each well to solubilize the blue formazan dye. Optical density (OD) at 570 nm was measured by the Safire2™ microplate reader (Tecan). The cells without irradiation exposure were used as the control. The cell viability of the treatment groups was expressed as the percentage of the control.

### 2.5. Cell Migration Assay

Cells were cultured in Culture-Insert 2 wells in *μ*-Dish 35 mm (Cat No: 81176, ibidi, Germany) with a defined 500 *μ*m cell-free gap. The silicone inserts were carefully removed at 24 h after radiation exposure with doses ranging from 0.2 Gy to 5 Gy, and the dishes were observed under an inverted microscope (Nikon Instruments Inc., New York, USA). The images were taken with a DS-Fi3 Microscopy Camera with DS-L4 Tablet Interface (Nikon Instruments Inc., New York, USA). The gap distances were recorded at different time points. At 6 h after the removal of inserts, the results were used for comparison.

### 2.6. Immunofluorescence and Imaging of Cellular Junctions

HUVECs were cultured on Nunc™ Lab-Tek™ Chambered Coverglass (Cat No: 155383, Thermo Fisher Scientific, USA), which was coated with 100 *μ*l of 40 *μ*g/ml collagen (diluted in sterile water) for 15 min and washed with sterile water for three times. 24 h after exposure to 5 Gy gamma irradiation, the slides were fixed with 4% paraformaldehyde for 15 min and then treated with 0.5% Triton X-100 for 5 min at room temperature. After 3 washes with PBS, the cells were blocked with 1% bovine serum albumin (BSA) for 1 h and followed by incubation in the mouse antibody against occludin or VE-cadherin (1 : 200 in 1% BSA) overnight at 4°C. Subsequently, cells were incubated with Alexa Fluor 488- or Alexa Fluor 555-conjugated goat anti-mouse IgG in PBS containing 1% BSA for 1 h at room temperature. The slides were then applied with antifade mountant with DAPI, and the images were captured under a Leica florescence microscope (Leica Biosystems, Wetzlar, Germany).

### 2.7. Reverse Transcription-Quantitative Polymerase Chain Reaction (RT-qPCR)

Total RNA from HUVECs were extracted using an RNeasy Mini Kit according to the manufacturer's instructions. cDNA was synthesized using a Maxima First Strand cDNA Synthesis kit. 2 *μ*g RNA was mixed with 2 *μ*l Maxima Enzyme Mix, and the 4 *μ*l reaction mix (5x) contains the remaining reaction components: reaction buffer, dNTPs, oligo(dT)18, and random hexamer primers, in total a 20 *μ*l reaction volume. cDNA synthesis reaction was performed at 25°C for 10 min, 50°C for 30 min, and 85°C for 5 min. Real-time PCR was performed in triplicate in the Applied Biosystems™ QuantStudio 6 Flex Real-Time PCR System (Thermo Fisher Scientific, Singapore). A final 20 *μ*l volume included 2 *μ*l cDNA; 10 *μ*l Maxima SYBR Green/ROX qPCR Master Mix (2x) containing Maxima Hot Start Taq DNA Polymerase, SYBR Green I, ROX passive reference dye, and dNTPs (also dUTP) in an optimized PCR buffer; and 1 *μ*M each of the respective forward and reverse primers. Reactions were performed at 50°C for 2 min and 95°C for 10 min, 40 cycles of denaturation at 95°C for 15 s, and annealing and extension at 60°C for 30 s and 72°C for 30 s. The amount of the target gene was normalized to GAPDH and was calculated by the 2^−ΔΔCT^ method. Results were expressed as the fold change to the control group. The primers used for real-time PCR are listed in Table 1.

### 2.8. Immunoblotting Analysis

HUVECs were cultured and harvested by scraping and centrifugation, washed twice with ice-cold PBS, and lysed in CelLytic™ mammalian cell lysis/extraction reagent containing protease and a phosphatase inhibitor cocktail. After incubation in ice for 20 minutes with agitation, supernatants were collected by centrifugation at 15,000 × *g* for 15 min. Protein concentration was determined using a BCA assay kit. Protein lysates were separated by gel electrophoresis of 10%-12% SDS-PAGE and then transferred to a nitrocellulose membrane. The membranes were blocked by 5% BSA and incubated with the respective primary antibodies (*β*-actin, p65, and p53 at 1 : 1000 dilution; ZO-1, occludin, VE-cadherin, connexin 40, eNOS, p-p38, p38, p21, p27, and p-p65 at 1 : 500 dilution) overnight at 4°C and HRP-conjugated secondary antibodies (1 : 10,000 dilution) at room temperature for 1 h. Immunoreactive proteins were then visualized by the ECL method, and the image was captured and quantified by the Bio-Rad Gel Doc system. *β*-Actin served as the loading control. Band densities from different time points were measured by ImageJ and normalized to the respective loading control. The fold change relative to the control group was calculated.

### 2.9. Measurement of ROS

Fluorescent probe H_2_DCFDA was used to measure the intracellular level of ROS. H_2_DCFDA was a cell-permeant and nonfluorescent probe, which was converted to the fluorescent 2′,7′-dichlorofluorescein (DCF) when intracellular esterases and oxidation cleaved the acetate groups. Briefly, HUVECs were seeded in 60 mm^2^ cell culture dishes. At 6 h after radiation exposure to different doses, cells were stained with 25 *μ*M H_2_DCFDA in PBS at 37°C for 30 min in the dark and then harvested by trypsinization. HUVECs were resuspended in PBS and subjected to flow cytometry (BD LSRFortessa, BD Biosciences, USA) at excitation and emission wavelengths of 485 nm and 535 nm, respectively. The fluorescence intensities were used for comparison.

### 2.10. NO Production by the Griess Reagent System

NO_2_^−^ concentration was measured by the Griess Reagent. HUVECs were exposed to gamma irradiation at different doses. At 6 h postirradiation, the supernatant medium was collected. 50 *μ*l sample or nitrite standard, 50 *μ*l sulfanilamide solution, and 50 *μ*l NED solution were added into a 96-well plate and incubated for 5-10 min at room temperature in the dark. After a purple/magenta color formed, the absorbance at the 520 nm wavelength was measured within 30 min in a plate reader (Tecan Infinite F200 Pro, Switzerland).

### 2.11. Levels of Inflammatory Factors IL-6 and TNF-*α* by RT-qPCR and ELISA

mRNA expression of IL-6 and TNF-*α* was measured by RT-qPCR as described above. The protein levels of IL-6 and TNF-*α* in the HUVEC lysate were measured using ELISA. 100 *μ*l standard or sample solutions were added to a 96-well plate, which was coated by the capture antibodies and blocked by 300 *μ*l Reagent Diluent. After incubation for 2 h at room temperature, the plate was washed and the detection antibody was added. A working solution of streptavidin-HRP, substrate, and stop solution was added sequentially. The optical density of each well was measured at 450 nm using a microplate reader (Tecan Infinite F200 Pro, Switzerland). IL-6 and TNF-*α* protein levels in different irradiation groups were calculated according to the respective standard curve.

### 2.12. The Activation of MAPK/NF-*κ*B Pathways Evaluated by Western Blot

The expression of MAPK/NF-*κ*B pathway-related proteins was evaluated by western blot as described above.

### 2.13. Nuclear and Cytoplasmic Fraction Extraction

After washing with ice-cold PBS with phosphatase inhibitors, HUVECs were gently removed by scraping. After centrifugation at 500 rpm for 5 min, the cell pellet was resuspended in 1x hypotonic buffer by pipetting up and down several times and adding 25 *μ*l detergent. The mixture was centrifuged at 14,000 × *g* for 30 s, and the cytoplasmic fraction in the supernatant was collected and stored at -80°C. The nuclear pellet was resuspended in complete lysis buffer, vortexed, and incubated on ice for 30 min. After centrifugation at 14,000 × *g* for 10 min, the supernatant containing the nuclear fraction was collected and stored at -80°C.

### 2.14. NF-*κ*B DNA-Binding Activity by TransAM™ NF-*κ*B Transcription Factor Assay Kit

The TransAM™ NF-*κ*B Transcription Factor Assay kit was used to determine the NF-*κ*B DNA-binding activity according to the manufacturer's instruction. Briefly, each well in the plate was added with 30 *μ*l complete binding buffer and 20 *μ*l sample diluted in a complete lysis buffer. In the blank wells, sample solution was replaced by 20 *μ*l complete lysis buffer. The reaction was incubated with mild agitation (100 rpm) at room temperature for 1 h. After washing 3 times, 100 *μ*l diluted NF-*κ*B antibody was added and incubated at room temperature for 1 h without agitation. The plate was washed and then added by HRP-conjugated antibody, substrate and stop solutions sequentially. The absorbance was read at 450 nm on a microplate reader within 5 min.

### 2.15. BAY 11-7085 Treatment on the Barrier Function Examined by Electric Cell-Substrate Impedance Sensing (ECIS) and FITC-Dextran Permeability Assay

BAY 11-7085 was dissolved in DMSO at the concentration of 10 mM as stock and diluted to the final concentration of 2.5 *μ*M with the cell medium before the experiments.

### 2.16. Barrier Function of Cell Monolayers Monitored Using ECIS

An ECIS Z*θ* instrument (Applied BioPhysics, Troy, NY, USA) was used in this study to measure transendothelial electrical resistance (TEER) in HUVECs. These real-time electrode arrays were performed to monitor electrical resistance, impedance, and capacitance across cells. The ECIS program was set to measure electrical resistance at a single frequency of 4000 Hz. 8W10E+ arrays were selected for our experiments. This array had 8 wells. Each contained two sets of 20 gold-film electrodes interdigitated for bioelectrical measurements across cell layers. The array was coated with collagen as described above and then 200 *μ*l medium was added for electrode stabilization. HUVECs were added to two coated arrays, and the arrays were kept in a 5% CO_2_ humidified incubator at 37°C during the resistance measurements. BAY 11-7085 was added to one 8W10E+ array one hour before gamma irradiation with 5 Gy, and the other without irradiation was used as the control. TEER was continuously measured.

### 2.17. FITC-Dextran Permeability Assay

6.5 mm Transwell inserts with 0.4 *μ*m pore polyester membrane placed in 24-well plates (Cat No: 3470, Corning, USA) were coated with collagen as described above. HUVECs were seeded and reached complete confluence, and then BAY 11-7085 at designed concentrations was added into the inserts. One hour later, the inserts were exposed to 5 Gy gamma irradiation. Nonirradiated inserts were used as the control. 24 h postirradiation, 200 *μ*l FITC-dextran of 4 kDa at a final concentration of 1 mg/ml was added into the upper inserts and 1 ml medium was added into the lower chambers. The plate was incubated at 37°C for 1 hour and 30 minutes. Inserts were removed and 100 *μ*l medium from the lower chamber was collected and transferred into a 96-well plate with black walls and bottoms. The florescence intensity was measured at excitation and emission wavelengths of 492 nm and 518 nm, respectively. Changes in fluorescence intensity were calculated and compared among different groups.

### 2.18. Statistical Analysis

Values of the normal distribution were expressed as the mean ± standard error of the mean (SEM). One-way analysis of variance (ANOVA) was used to determine statistical significance between groups followed by Tukey's post hoc test. A probability value of *p* < 0.05 was taken as a statistical significance.

## 3. Results

### 3.1. Gamma Irradiation Attenuated Migration Ability in HUVECs but Not Cell Viability

**Figure 1:**
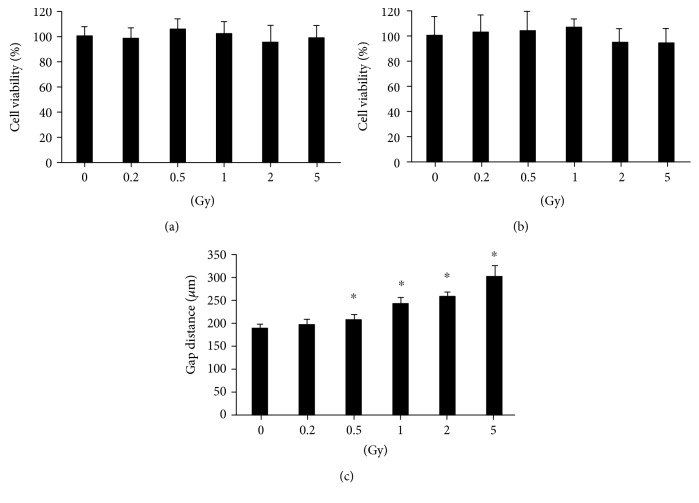
Cell viability and migration ability in HUVECs after gamma irradiation of different doses. Gamma irradiation with doses ranging from 0.2 Gy to 5 Gy does not induce HUVEC viability changes when measured at 6 h (a) and 24 h (b) postirradiation by the MTT method. However, it increased the gap distance when HUVECs were irradiated with radiation doses ranging from 0.5 to 5 Gy (c). The cell viability of the treatment groups is expressed as the percentage of the control; cell migration is measured by gap closure test. ^∗^A value of *p* < 0.05 is taken as statistical significance.

The cell viability in HUVECs was tested after radiation exposure ranging from 0.2 to 5 Gy. No significant decrease was observed at 6 h (Figure 1(a)) and 24 h (Figure 1(b)) postirradiation. However, the gap distance was increased significantly after gamma radiation with doses ranging from 0.5 to 5 Gy (Figure 1(c)), indicating that gamma irradiation attenuated the migration ability of HUVECs.

### 3.2. Gamma Irradiation Caused Cellular Junction Damage in HUVECs

**Figure 2:**
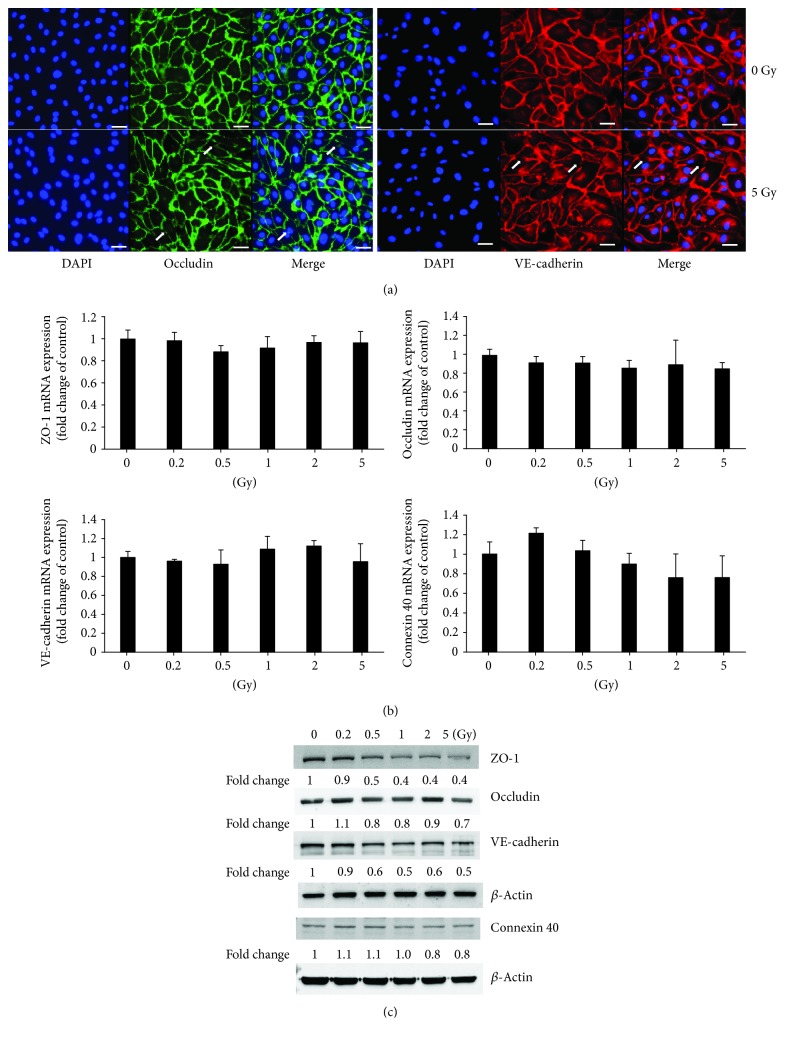
Cellular junction examination in HUVECs after exposure to gamma irradiation. Immunofluorescence examination indicates the damage of the cellular barrier 24 h after irradiation with 5 Gy (the impaired and disconnected junctions between two adjoined cells pointed by arrows in the lower panels in (a)) (×400). Scale bar: 50 *μ*m. While the gene expressions of junction-related molecules such as ZO-1, occludin, VE-cadherin, and connexin 40 do not change significantly (b), there are obvious reductions of protein expressions of ZO-1, occludin, VE-cadherin, and connexin 40 (c).

Immunocytochemical analysis showed cellular junctions between adjacent cells characterized by bright fluorescence (green for occludin and red for VE-cadherin). The nucleus was stained by DAPI. HUVECs with 5 Gy radiation exposure exhibited the impaired and disconnected intercellular junctions indicated by the visible disruption of labeling (pointed by arrows) (Figure 2(a)). Western blot results showed the decreased expression of junction-related proteins such as ZO-1, occludin, VE-cadherin, and connexin 40 in HUVECs after gamma irradiation (Figure 2(c)), although their mRNA expression did not change significantly (Figure 2(b)).

### 3.3. Gamma Irradiation-Induced Oxidative and Nitrosative Stress in HUVECs

**Figure 3:**
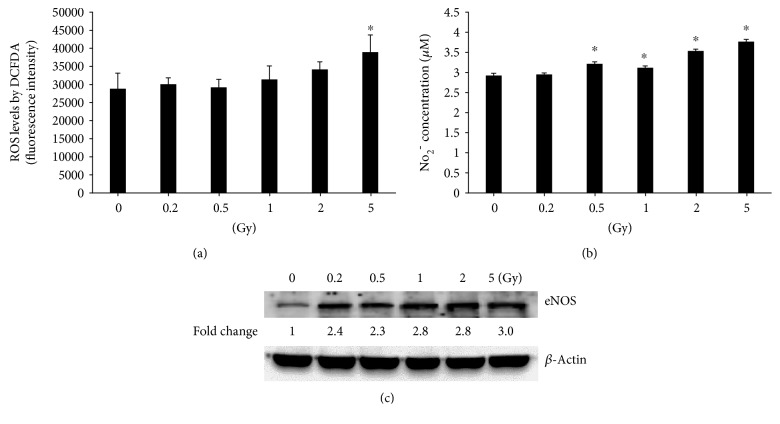
Gamma irradiation induces oxidative and nitrosative stresses in HUVECs. The intracellular ROS is increased significantly 6 h after irradiation with 5 Gy (a). NO_2_^−^ concentration is increased significantly 6 h after irradiation with doses from 0.5 to 5 Gy (b). Western blot shows increased eNOS after irradiation with doses from 0.2 to 5 Gy (c). ^∗^A value of *p* < 0.05 is taken as statistical significance.

Gamma irradiation with 5 Gy significantly increased ROS levels in HUVECs when compared with the nonirradiated control (Figure 3(a)), while the NO_2_^−^ concentration was higher in the HUVEC medium after radiation exposure with doses ranging from 0.5 Gy to 5 Gy (Figure 3(b)). This enhancement was accompanied by the increased protein levels of eNOS in HUVECs after gamma irradiation (Figure 3(c)).

### 3.4. Gamma Irradiation Promoted Gene and Protein Expression of Inflammatory Cytokines in HUVECs

**Figure 4:**
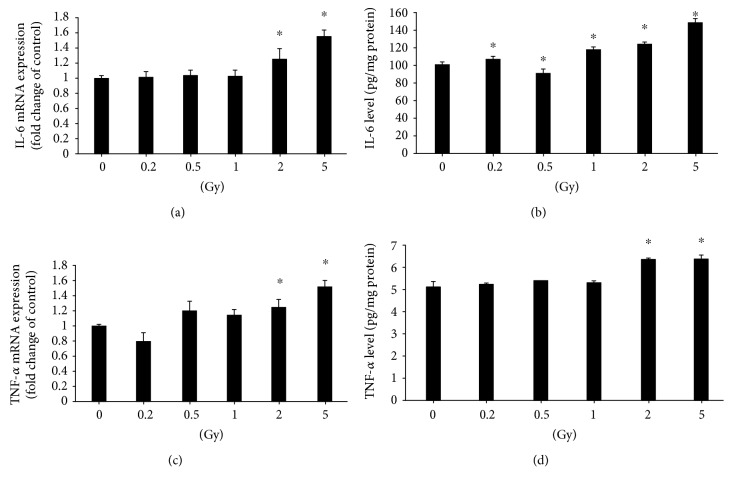
Gamma irradiation induces gene and protein expressions of cytokines. Irradiation with 2 or 5 Gy induces upregulation of the IL-6 gene 6 h after irradiation (a), but upregulation of the IL-6 protein is induced by irradiation with doses ranging from 0.2 to 5 Gy (b). Both gene (c) and protein (d) expressions of TNF-*α* are upregulated after irradiation with 2 or 5 Gy. ^∗^A value of *p* < 0.05 is taken as statistical significance.

The mRNA expression of inflammatory cytokines IL-6 and TNF-*α* was increased in HUVECs after radiation exposure with doses of 2 Gy and 5 Gy (Figures 4(a) and 4(c)). ELISA results demonstrated that the IL-6 protein level was significantly higher in HUVECs after radiation exposures with doses ranging from 0.2 Gy to 5 Gy (Figure 4(b)) when compared to the control, while the TNF-*α* protein level was increased after gamma radiation with 2 Gy and 5 Gy (Figure 4(d)).

### 3.5. Gamma Irradiation Increased the Protein Levels of MAPK Pathway Molecules and the Activation of the NF-*κ*B Pathway

**Figure 5:**
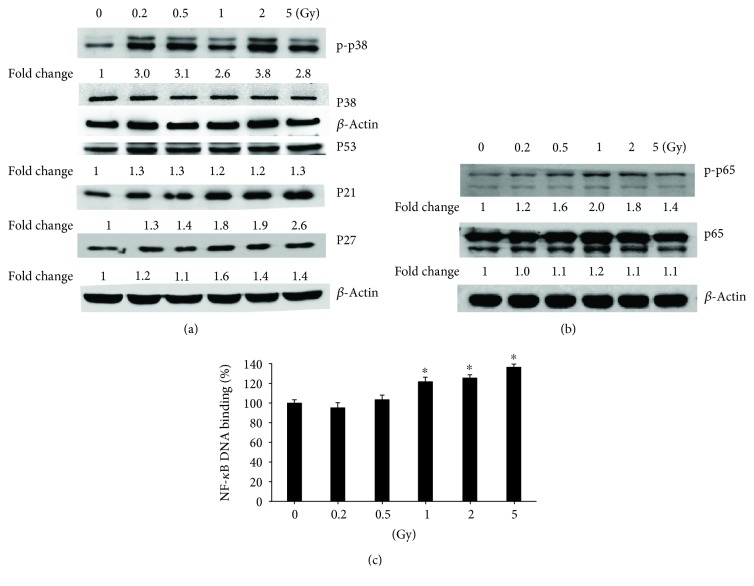
Gamma irradiation activates MAPK/NF-*κ*B pathways. (a) The activation of MAPK pathway molecules p-p38, p38, p53, p21, and p27 in HUVECs after exposure to gamma irradiation was measured by western blot. (b) Protein levels of p-p65 and p65 were examined by western blot. (c) NF-*κ*B DNA-binding activity by TransAM™ NF-*κ*B Transcription Factor Assay kit. The absorbance at 450 nm was read on a microplate reader. Values were expressed as mean ± S.E.M. One-way analysis of variance (ANOVA) was used to determine statistical significance between groups followed by Tukey's post hoc test. ^∗^A value of *p* < 0.05 was taken as statistical significance.

Western blot results showed that the protein levels of MAPK pathway molecules p-p38, p53, p21, and p27 increased in HUVECs after exposure to gamma irradiation (Figure 5(a)). The enhancement further induced the activation of the NF-*κ*B pathway, indicated by the higher protein levels of p-p65 (Figure 5(b)) and increased NF-*κ*B DNA-binding activity (Figure 5(c)).

### 3.6. BAY 11-7085 Treatment on the Barrier Function Examined by ECIS and FITC-Dextran Permeability Assay

**Figure 6:**
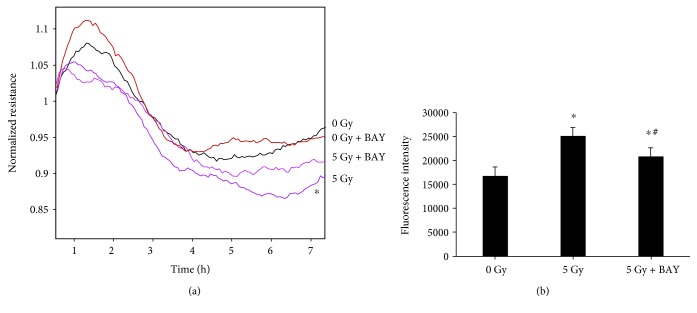
BAY 11-7085 treatment on the barrier function examined by ECIS and FITC-dextran permeability assay. Irradiation with 5 Gy significantly reduces the resistance in HUVECs when compared to the nonirradiated (0 Gy) group (a). The treatment with BAY 11-7085 at 2.5 *μ*M partially blocked the decreased resistance in HUVECs caused by 5 Gy gamma irradiation, while the treatment alone did not have any significant effects in HUVECs without gamma radiation exposure (a). The fluoresce intensity of FITC-labelled dextrans (4 kDa) was significantly higher after 5 Gy gamma irradiation, and the treatment of BAY 11-7085 partially inhibited this increase (b). ^∗^*p* < 0.05 vs. 0 Gy; ^#^*p* < 0.05 vs. 5 Gy.

The effect of 5 Gy gamma radiation on the barrier function of HUVECs was assessed by TEER measurements. A higher resistance indicates a tighter membrane barrier. After the postirradiation stabilization of arrays, we observed that irradiation with 5 Gy significantly decreased the resistance in HUVECs when compared with the nonirradiated group (0 Gy), indicating the barrier disruption in HUVECs after gamma irradiation (Figure 6(a)). BAY 11-7085, as an inhibitor of NF-*κ*B activation and phosphorylation of I*κ*B*α*, was found to partially block the decrease of TEER in HUVECs after 5 Gy gamma irradiation. No significant influence on TEER was observed when BAY 11-7085 was applied alone at 2.5 *μ*M in HUVECs without irradiation (Figure 6(a)).

It is believed that TEER is used to determine the ion permeability of cellular junctions, and fluorescently labelled compounds of different sizes are suitable for monitoring the paracellular permeability of hydrophilic tracers. In this study, the fluoresce intensity of FITC-labelled dextrans (4 kDa) was found significantly higher in the lower chamber medium after 5 Gy gamma irradiation, while the application of BAY 11-7085 partially inhibited this increase (Figure 6(b)).

## 4. Discussion

Endothelial cells play an important role in maintaining the intercellular barrier, which controls the passage of macromolecules, proteins, and circulating cells between the interstitial tissues and blood. The damage of the endothelial barrier and its integrity is associated with the development of CVD complications. An increase in vascular permeability is an immediate cellular damage following radiation, which further impairs vascular functions and enhances cardiovascular risk [9, 28, 29]. Our study demonstrated that gamma irradiation with doses ranging from 0.5 to 5 Gy dose-dependently inhibited the migratory ability of HUVECs without cell viability change at 6 h and 24 h post exposure. Moreover, 5 Gy irradiation exposure also caused the disruption of intercellular junctions, decreased TEER, and increased permeability in HUVECs in this study. These results demonstrated that gamma irradiation with the doses applied in our study significantly influences the intercellular barrier function without changing the number of cells. Previous observation also showed that single and fractionated low-dose irradiations could induce chronic vascular inflammation and further stimulate the process of atherosclerosis but do not influence cell viability and DNA repair [9]. Gabrys et al. reported that radiation caused a rapid and persistent increase in permeability as well as F-actin and VE-cadherin remodelling in microvascular cells, but actin remodelling was not observed in HUVECs though they are equally radiosensitive [3]. This discrepancy might be due to the differences in radiation sources between X-rays and gamma rays or the endpoints between 30 min to 6 h and 24 h postirradiation. The permeability assay by dextran might be insufficiently sensitive to detect the barrier function change. We did demonstrate the resistance alteration at 6 h postirradiation examined by ECIS. ECIS is a very sensitive technology to record slight changes in TEER and has the potential to continuously detect radiation-induced barrier changes in HUVECs. It has been demonstrated that after irradiation of HUVECs to doses as low as 2 Gy, TEER was reduced at 3 h, indicating that gamma radiation induces an early disruption in the endothelial barrier at lower doses than those required for cytotoxic effects [11].

Several endpoints were selected in this study from 0.2 Gy to 5 Gy gamma irradiation. The dose-dependent effects were observed in migration ability, oxidative and nitrosative stresses, inflammation, and NF-*κ*B DNA-binding activity in our results, but not in cell viability, mRNA and protein levels of cellular junction-related genes, and MAPK/NF-*κ*B pathway molecules. Some epidemiological studies suggested a linear dose-response relationship for radiation risks; e.g., one report showed statistically significant risks for leukaemia in over 110,000 Chernobyl cleanup workers [30]. Even several data points below 100 mSv showed a linear risk increase [30]. Other studies also supported the linear no-threshold theory of radiation risks, e.g., the relationship of CT scans in childhood and subsequent risk of leukaemia and brain tumors [31] and the correlation of natural background radiation and the incidence of childhood leukaemia and other cancers [32]. However, much debate exists about the validity of the linear no-threshold theory for radiation risks, especially for the effects of low-dose radiation less than 100 mSv [33–35]. It has been debated whether they are beneficial or detrimental, and so far, there is still lack of consistency among the available experimental data. Our results showed no linear dose-response effects in most parameters in HUVEC cell lines after exposure to gamma irradiation, but it is hard to correlate radiation doses applied in in vitro experiments with those in clinical applications. The defense mechanisms, e.g., DNA repair or defenses against ROS, should be considered in evaluating the dose-response effects.

Endothelial layer integrity depends on the intercellular junctions including tight junctions, adherens junctions, and gap junctions [36]. The components of a tight junction include transmembrane and cytoplasmic proteins. The transmembrane proteins, such as claudins, occludins, and junctional adhesion molecules (JAMs), are linked to the actin cytoskeleton through cytoplasmic components such as ZO-1, ZO-2, and ZO-3 and a host of other associated signaling proteins [37]. The adherens junction is a cell junction in which the cytoplasmic face is linked to the actin cytoskeleton. VE-cadherin is an endothelial specific adherens junction protein [13]. It associates with the actin cytoskeleton via *α*-, *β*-, and *γ*-catenins and with RhoGAP via the p120-catenin [38]. Gap junctions form channels between the cytoplasm of adjacent cells and allow the pass-through of electrical impulses, various molecules, and ions. The core proteins of these channels are connexins [14]. Under pathologic conditions, inflammatory mediators may compromise the barrier function and induce active remodelling of the cytoskeleton and formation of gaps between adjacent endothelial cells. Zorkina et al. demonstrated a decrease in fluorescence intensity and mRNA expression of cellular junction proteins ZO-1, *β*-catenin, and connexin 43 after gamma radiation in HUVECs cocultured with allogeneic astrocytes [12]. The decreased expression of Cx43 after 20 Gy X-ray irradiation was also observed in human dermal lymphatic endothelial cells accompanied by the increased permeability [39]. Our study showed a decrease in the protein expression of ZO-1, occludin, VE-cadherin, and connexin 40 after gamma irradiation, leading to the impairment of the barrier function. However, no significant differences in mRNA expression of these molecules were observed. One study also indicated that exposure to ionizing radiation induced the barrier damage and increased the permeability of the blood-brain barrier but without massive modification of the tight junction proteins ZO-1, ZO-2, claudin-5, and occludin [17].

Gap junction channels and connexin regulation are also implicated in vascular disruption. The expression of connexins has been proved to increase the resistance of a variety of cells to cellular injuries including ionizing radiation. The forced expression of connexin 40, connexin 43, and connexin 32 increased the resistance to injury of oxidative stress and UV irradiation in C6 glioma [40]. Our results showed a decrease of mRNA expression of connexin 40 after gamma irradiation, though not statistically significant, accompanied by a minor decrease in protein levels of connexin 40. No previous reports have been published regarding the contribution of connexin 40 in HUVECs exposed to gamma irradiation. One study indicated that 5 Gy X-rays induced a significantly elevated level of connexin 43 in mouse endothelial cell line bEnd3 but downregulation of connexin 43 mRNA in human hybrid endothelial cells (EA.hy926), indicating a cell line-specific modulation of connexin 43 expression after exposure to X-rays [41]. Further investigations are still needed to clarify the function of gap junction channels and connexins implicated in gamma radiation-induced barrier injury.

Oxidative and nitrosative stresses and inflammatory response are all involved in radiation-induced pathogenesis [18, 19, 42]. Reactive free radicals and water radiolysis induce the structural and functional changes in cell junctions and intercellular communication, which further influence the endpoints of gamma radiation effects, e.g., cell death [43]. Ionizing irradiation exerts its biological effects via initially generating ROS, which reacts with unsaturated lipids, alters the membrane permeability, and induces the lipid peroxidation and the production of proinflammatory cytokines, such as TNF-*α*, IL-6, and IL-1*β* [19, 44]. It has been demonstrated that gamma radiation increased intracellular ROS levels and decreased antioxidant production in endothelial cells [45, 46], especially dose-dependently increased intracellular levels of ROS in HUVECs at 24 h postirradiation [47]. This is consistent with our observation showing elevated levels of ROS, NO, and eNOS, accompanied by the higher levels of diverse cytokines such as IL-6 and TNF-*α*. The increased cytokines such as IL-6, IL-8, monocyte chemoattractant protein 1 (MCP-1), granulocyte-colony stimulating factor (G-CSF), and other proinflammatory cytokines were also demonstrated in other studies in HUVECs after radiation exposure [48]. These results suggest that gamma irradiation-induced oxidative and nitrosative stresses and inflammatory cytokines in the endothelium are involved in the genesis of different vascular diseases.

The initiation of endothelial cell injury in response to radiation is concomitant with the activation of stress-activated kinase cascades, e.g., MAPK/NF-*κ*B [49]. MAPK includes extracellular signal-regulated kinase 1/2 (Erk1/2), c-Jun N-terminal kinase (JNK), and p38. Sustained activation of JNK, Erk1/2, and/or p38 is responsible for the transduction of a radiation damage signal. p38 MAPK was demonstrated to predominantly mediate gamma irradiation-induced endothelial cell apoptosis [49, 50]. p38 MAPK and NF-*κ*B activation was observed 24 h after 2 Gy gamma irradiation in HUVECs [51]. The modulation of the NF-*κ*B pathway has been believed to contribute to the survival of the irradiated cells [52]. A study demonstrated that radiation-induced apoptosis is mediated by NF-*κ*В signaling in primary bovine aortic endothelial cells [53]. We observed the increased expression of p-p38, p53, p27, p21, p-p65, and NF-*κ*B DNA-binding activities in HUVECs after gamma irradiation. To further validate our hypothesis that gamma irradiation-induced injury in the cellular barrier of HUVECs was through the MAPK/NF-*κ*B inflammatory pathway, BAY 11-7085, an inhibitor of NF-*κ*B activation and phosphorylation of I*κ*B*α*, was employed in this study. The treatment of HUVECs with BAY 11-7085 partially blocked the decrease of TEER and increased permeability after 5 Gy gamma irradiation. Our observation not only demonstrated the involvement of the NF-*κ*B inflammatory pathway in gamma radiation-induced disruption of intercellular barriers but also pointed to a possibility that other signaling pathways may contribute to this damage too. One report indicated that the activation of the STAT3 pathway was involved in radiation-induced endothelial inflammation [54], and the DNA-binding activity of STAT3 and the level of phospho-Stat3 Tyr (705), but not phospho-Stat3 Ser (727), was reduced in HUVECs after 3 Gy irradiation [55]. The contribution of the STAT3 pathway to the gamma radiation-induced disruption of the cellular barrier is not investigated in our experiments. This is the limitation and also the future work of our study.

In summary, our results demonstrated that gamma irradiation inhibited the migration ability, decreased TEER, increased permeability, and induced disruption of cellular junctions in HUVECs accompanied by the lower levels of junction-related proteins such as ZO-1, occludin, VE-cadherin, and connexin 40. The enhanced oxidative and nitrosative stresses, e.g., ROS and NO^2-^ levels, inflammatory cytokines IL-6 and TNF-*α*, and the activation of the MAPK/NF-*κ*B signaling pathway contributed to gamma irradiation-induced damage, which was partially inhibited by the treatment of the NF-*κ*B activation inhibitor BAY 11-7085. We therefore concluded that the gamma irradiation-induced disruption of cellular junctions in HUVECs was through the inflammatory MAPK/NF-*κ*B signaling pathway.

## Figures and Tables

**Table 1:** 

Gene	Forward	Reverse
ZO-1	AGCCATTCCCGAAGGAGTTG	GCAAAAGACCAACCGTCAGG
Occludin	ACTTCAGGCAGCCTCGTTAC	CCTGATCCAGTCCTCCTCCA
VE-cadherin	ATGTAGGCAAGATCAAGTCAAG	CCTCTCAATGGCGAACAC
Connexin 40	CCATGGAGGTGGGCTTCATT	AGGCTAAGGAGGAGGGACAG
IL-6	AATGAGGAGACTTGCCTGGT	GCAGGAACTGGATCAGGACT
TNF-*α*	TCTTCTCGAACCCCGAGTGA	TATCTCTCAGCTCCACGCCA
GAPDH	GCACCGTCAAGGCTGAGAAC	TGGTGAAGACGCCAGTGGA

## Data Availability

All the data could be provided at the request of the readers.

## References

[1] Schollnberger H., Eidemuller M., Cullings H. M., Simonetto C., Neff F., Kaiser J. C. (2018). Dose-responses for mortality from cerebrovascular and heart diseases in atomic bomb survivors: 1950–2003. *Radiation and Environmental Biophysics*.

[2] Gillies M., Richardson D. B., Cardis E. (2017). Mortality from circulatory diseases and other non-cancer outcomes among nuclear workers in France, the United Kingdom and the United States (INWORKS). *Radiation Research*.

[3] Gabrys D., Greco O., Patel G., Prise K. M., Tozer G. M., Kanthou C. (2007). Radiation effects on the cytoskeleton of endothelial cells and endothelial monolayer permeability. *International Journal of Radiation Oncology, Biology, Physics*.

[4] Young E. F., Smilenov L. B. (2011). Impedance-based surveillance of transient permeability changes in coronary endothelial monolayers after exposure to ionizing radiation. *Radiation Research*.

[5] Ungvari Z., Podlutsky A., Sosnowska D. (2013). Ionizing radiation promotes the acquisition of a senescence-associated secretory phenotype and impairs angiogenic capacity in cerebromicrovascular endothelial cells: role of increased DNA damage and decreased DNA repair capacity in microvascular radiosensitivity. *The Journals of Gerontology: Series A*.

[6] Yentrapalli R., Azimzadeh O., Barjaktarovic Z. (2013). Quantitative proteomic analysis reveals induction of premature senescence in human umbilical vein endothelial cells exposed to chronic low-dose rate gamma radiation. *Proteomics*.

[7] Park M. T., Oh E. T., Song M. J., Lee H., Park H. J. (2012). Radio-sensitivities and angiogenic signaling pathways of irradiated normal endothelial cells derived from diverse human organs. *Journal of Radiation Research*.

[8] Mancuso M., Pasquali E., Braga-Tanaka I. (2015). Acceleration of atherogenesis in *ApoE^−/−^* mice exposed to acute or low-dose-rate ionizing radiation. *Oncotarget*.

[9] Cervelli T., Panetta D., Navarra T. (2014). Effects of single and fractionated low-dose irradiation on vascular endothelial cells. *Atherosclerosis*.

[10] Kumarathasan P., Vincent R., Blais E. (2013). Cardiovascular changes in atherosclerotic ApoE-deficient mice exposed to Co60 (*γ*) radiation. *PLoS One*.

[11] Sharma P., Templin T., Grabham P. (2013). Short term effects of gamma radiation on endothelial barrier function: uncoupling of PECAM-1. *Microvascular Research*.

[12] Zorkina Y. A., Volgina N. E., Gorlachev G. E. (2014). Effect of *γ*-irradiation on expression of tight and adherens junction protein mRNA on *in vitro* blood–brain barrier model. *Bulletin of Experimental Biology and Medicine*.

[13] Kabacik S., Raj K. (2017). Ionising radiation increases permeability of endothelium through ADAM10-mediated cleavage of VE-cadherin. *Oncotarget*.

[14] Azzam E. I., de Toledo S. M., Little J. B. (2003). Expression of *CONNEXIN43* is highly sensitive to ionizing radiation and other environmental stresses. *Cancer Research*.

[15] Ivanova E., Kovacs-Oller T., Sagdullaev B. T. (2019). Domain-specific distribution of gap junctions defines cellular coupling to establish a vascular relay in the retina. *The Journal of Comparative Neurology*.

[16] Zhou H. S., Li M., Sui B. D. (2018). Lipopolysaccharide impairs permeability of pulmonary microvascular endothelial cells via connexin40. *Microvascular Research*.

[17] Fauquette W., Amourette C., Dehouck M. P., Diserbo M. (2012). Radiation-induced blood-brain barrier damages: an in vitro study. *Brain Research*.

[18] Wang H., Sethi G., Loke W. K., Sim M. K. (2015). Des-aspartate-angiotensin I attenuates mortality of mice exposed to gamma radiation via a novel mechanism of action. *PLoS One*.

[19] Wang H., Sim M. K., Loke W. K. (2017). Potential protective effects of ursolic acid against gamma irradiation-induced damage are mediated through the modulation of diverse inflammatory mediators. *Frontiers in Pharmacology*.

[20] Zhang J., Rane G., Dai X. (2016). Ageing and the telomere connection: an intimate relationship with inflammation. *Ageing Research Reviews*.

[21] Shanmugam M. K., Sethi G. (2013). Role of epigenetics in inflammation-associated diseases. *Sub-Cellular Biochemistry*.

[22] Morris G., Fernandes B. S., Puri B. K., Walker A. J., Carvalho A. F., Berk M. (2018). Leaky brain in neurological and psychiatric disorders: drivers and consequences. *Australian & New Zealand Journal of Psychiatry*.

[23] Yu H., Aravindan N., Xu J., Natarajan M. (2017). Inter- and intra-cellular mechanism of NF-*κ*B-dependent survival advantage and clonal expansion of radio-resistant cancer cells. *Cellular Signalling*.

[24] Li F., Sethi G. (2010). Targeting transcription factor NF-*κ*B to overcome chemoresistance and radioresistance in cancer therapy. *Biochimica et Biophysica Acta (BBA) - Reviews on Cancer*.

[25] Sethi G., Tergaonkar V. (2009). Potential pharmacological control of the NF-*κ*B pathway. *Trends in Pharmacological Sciences*.

[26] Vu H. T., Kotla S., Ko K. A. (2018). Ionizing radiation induces endothelial inflammation and apoptosis via p90RSK-mediated ERK5 S496 phosphorylation. *Frontiers in Cardiovascular Medicine*.

[27] Olteanu D., Baldea I., Clichici S. (2014). In vitro studies on the mechanisms involved in chemoprevention using *Calluna vulgaris* on vascular endothelial cells exposed to UVB. *Journal of Photochemistry and Photobiology B, Biology*.

[28] Schultz-Hector S., Trott K. R. (2007). Radiation-induced cardiovascular diseases: is the epidemiologic evidence compatible with the radiobiologic data?. *International Journal of Radiation Oncology, Biology, Physics*.

[29] Krizak J., Frimmel K., Bernatova I., Navarova J., Sotnikova R., Okruhlicova L. (2016). The effect of omega-3 polyunsaturated fatty acids on endothelial tight junction occludin expression in rat aorta during lipopolysaccharide-induced inflammation. *Iranian Journal of Basic Medical Sciences*.

[30] Zablotska L. B., Bazyka D., Lubin J. H. (2013). Radiation and the risk of chronic lymphocytic and other leukemias among Chornobyl cleanup workers. *Environmental Health Perspectives*.

[31] Pearce M. S., Salotti J. A., Little M. P. (2012). Radiation exposure from CT scans in childhood and subsequent risk of leukaemia and brain tumours: a retrospective cohort study. *The Lancet*.

[32] Kendall G. M., Little M. P., Wakeford R. (2013). A record-based case–control study of natural background radiation and the incidence of childhood leukaemia and other cancers in Great Britain during 1980–2006. *Leukemia*.

[33] Cardarelli J. J., Ulsh B. A. (2018). It is time to move beyond the linear no-threshold theory for low-dose radiation protection. *Dose-Response*.

[34] Tubiana M., Feinendegen L. E., Yang C., Kaminski J. M. (2009). The linear no-threshold relationship is inconsistent with radiation biologic and experimental data. *Radiology*.

[35] Seong K. M., Seo S., Lee D. (2016). Is the linear no-threshold dose-response paradigm still necessary for the assessment of health effects of low dose radiation?. *Journal of Korean Medical Science*.

[36] Guo R., Sakamoto H., Sugiura S., Ogawa M. (2007). Endothelial cell motility is compatible with junctional integrity. *Journal of Cellular Physiology*.

[37] Shukla P. K., Gangwar R., Manda B. (2016). Rapid disruption of intestinal epithelial tight junction and barrier dysfunction by ionizing radiation in mouse colon in vivo: protection by N-acetyl-l-cysteine. *American Journal of Physiology Gastrointestinal and liver Physiology*.

[38] Huo Z., Kong Y., Meng M., Cao Z., Zhou Q. (2019). Atorvastatin enhances endothelial adherens junctions through promoting *VE-PTP* gene transcription and reducing VE-cadherin-Y731 phosphorylation. *Vascular Pharmacology*.

[39] Kishimoto M., Akashi M., Kakei Y. (2018). Ionizing radiation enhances paracellular permeability through alteration of intercellular junctions in cultured human lymphatic endothelial cells. *Lymphatic Research and Biology*.

[40] Lin J. H., Yang J., Liu S. (2003). Connexin mediates gap junction-independent resistance to cellular injury. *The Journal of Neuroscience*.

[41] Banaz-Yasar F., Tischka R., Iliakis G., Winterhager E., Gellhaus A. (2005). Cell line specific modulation of connexin43 expression after exposure to ionizing radiation. *Cell Communication & Adhesion*.

[42] Berbee M., Fu Q., Boerma M. (2011). Reduction of radiation-induced vascular nitrosative stress by the vitamin E analog *γ*-tocotrienol: evidence of a role for tetrahydrobiopterin. *International Journal of Radiation Oncology, Biology, Physics*.

[43] Somosy Z., Horvath G., Bognar G., Koteles G. (2003). Structural and functional changes of cell junctions on effect of ionizing radiation. *Acta Biologica Szegediensis*.

[44] Sinha M., Das D. K., Manna K. (2012). Epicatechin ameliorates ionising radiation-induced oxidative stress in mouse liver. *Free Radical Research*.

[45] Yu J., Piao B. K., Pei Y. X., Qi X., Hua B. J. (2010). Protective effects of tetrahydropalmatine against gamma-radiation induced damage to human endothelial cells. *Life Sciences*.

[46] Yu J., Zhu X., Qi X., Che J., Cao B. (2013). Paeoniflorin protects human EA.hy926 endothelial cells against gamma-radiation induced oxidative injury by activating the NF-E2-related factor 2/heme oxygenase-1 pathway. *Toxicology Letters*.

[47] Hu S., Gao Y., Zhou H. (2017). New insight into mitochondrial changes in vascular endothelial cells irradiated by gamma ray. *International Journal of Radiation Biology*.

[48] Ebrahimian T., Le Gallic C., Stefani J. (2015). Chronic gamma-irradiation induces a dose-rate-dependent pro-inflammatory response and associated loss of function in human umbilical vein endothelial cells. *Radiation Research*.

[49] Kumar P., Miller A. I., Polverini P. J. (2004). p38 MAPK mediates *γ*-irradiation-induced endothelial cell apoptosis, and vascular endothelial growth factor protects endothelial cells through the phosphoinositide 3-kinase-Akt-Bcl-2 pathway. *Journal of Biological Chemistry*.

[50] Cary L. H., Noutai D., Salber R. E., Williams M. S., Ngudiankama B. F., Whitnall M. H. (2014). Interactions between endothelial cells and T cells modulate responses to mixed neutron/gamma radiation. *Radiation Research*.

[51] Soltani B., Bodaghabadi N., Mahpour G., Ghaemi N., Sadeghizadeh M. (2016). Nanoformulation of curcumin protects HUVEC endothelial cells against ionizing radiation and suppresses their adhesion to monocytes: potential in prevention of radiation-induced atherosclerosis. *Biotechnology Letters*.

[52] Manna K., Das U., Das D. (2015). Naringin inhibits gamma radiation-induced oxidative DNA damage and inflammation, by modulating p53 and NF-*κ*B signaling pathways in murine splenocytes. *Free Radical Research*.

[53] Natarajan M., Mohan S., Konopinski R., Otto R. A., Herman T. S. (2008). Induced telomerase activity in primary aortic endothelial cells by low-LET *γ*-radiation is mediated through NF-*κ*B activation. *The British Journal of Radiology*.

[54] Philipp J., Azimzadeh O., Subramanian V. (2017). Radiation-induced endothelial inflammation is transferred via the secretome to recipient cells in a STAT-mediated process. *Journal of Proteome Research*.

[55] Kim K. W., Mutter R. W., Cao C. (2006). Inhibition of signal transducer and activator of transcription 3 activity results in down-regulation of survivin following irradiation. *Molecular cancer therapeutics.*.

